# Identification of high risk and early stage eating disorders: first validation of a digital screening tool

**DOI:** 10.1186/s40337-021-00464-y

**Published:** 2021-09-06

**Authors:** Emma Bryant, Jane Miskovic-Wheatley, Stephen W. Touyz, Ross D. Crosby, Eyza Koreshe, Sarah Maguire

**Affiliations:** 1 InsideOut Institute for Eating Disorders, Faculty of Medicine and Health, Level 2, The Charles Perkins Centre, D17, The University of Sydney, Johns Hopkins Drive, Camperdown, NSW 2006 Australia; 2 Sanford Center for Biobehavioral Research, Sanford Research, Fargo, ND USA

**Keywords:** Eating disorders, Screening, Validation, Psychometrics, Anorexia, Bulimia

## Abstract

**Background:**

Eating disorders are amongst the deadliest of all mental disorders, however detection and early intervention rates remain extremely low. Current standardised screening questionnaires can be arduous or confronting and are ill-validated for online use, despite a universal shift to digital healthcare. The present study describes the development and pilot validation of a novel digital screening tool (the InsideOut Institute-Screener) for high risk and early stage eating disorders to drive early intervention and reduced morbidity.

**Methods:**

We utilised a mixed cross-sectional and repeated measures longitudinal survey research design to assess symptom severity and recognised parameters of statistical validity. Participants were recruited through social media and traditional advertising, and through MTurk. An Eating Disorders Examination Questionnaire (EDE-Q) global score of 2.3 and assessment of eating disorder behaviours was used to determine probable ED. 1346 participants aged 14–74 (mean [SE] age 26.60 [11.14] years; 73.8% female, 22.6% male) completed the survey battery. 19% were randomised to two-week follow-up for reliability analysis.

**Results:**

Strong positive correlations between the IOI-S and both the EDE-Q global (*r*_*s*_ = .88) and SCOFF (*r*_*s*_ = .75) total score were found, providing support for the concurrent validity of the scale. Inter-item correlations were moderate to strong (*r*_*s*_ = .46–.73). Correlations between the IOI-S and two measures of social desirability diverged, providing support for the discriminant validity of the scale. The IOI-S demonstrated high internal consistency (α = .908, ω = .910) and excellent two-week test–retest reliability (.968, 95% CI 0.959–0.975; *p* ≤ 0.1). The IOI-S accurately distinguished probable eating disorders (sensitivity = 82.8%, specificity = 89.7% [AUC = .944], LR^+^  = 8.04, LR^−^ = 0.19) and two stepped levels of risk.

**Conclusions and relevance:**

The present study provides excellent initial support for the psychometric validity of the InsideOut Institute digital screening tool, which has the potential to streamline early intervention in the hopes of reducing current high morbidity and mortality. Further validation should be undertaken in known clinical populations.

**Plain English Summary:**

Eating disorders are amongst the deadliest of all mental disorders, however detection and early intervention rates remain extremely low. The present study describes the initial psychometric validation of a novel digital screening tool (the InsideOut Institute Screener) for high risk and early stage eating disorders, for self-referral and/or use in primary care. 1346 participants aged 14–74 of all genders completed a survey battery designed to assess common parameters of statistical validity. Strong support was found for the screener’s ability to accurately measure eating disorder risk and symptomatology. The screener was highly positively correlated with a well known and extensively validated long form self-report questionnaire for eating disorder symptomatology. This study is a pilot validation and the genesis of a project that aims ultimately to drive early intervention leading to reduced morbidity and mortality rates in this illness group.

**Supplementary Information:**

The online version contains supplementary material available at 10.1186/s40337-021-00464-y.

## Introduction

Early intervention greatly improves prognostic outcomes for all of the major mental disorders including eating disorders. Despite the immense burden an eating disorder (ED) has on an individual, carers, and the healthcare system, detection in primary care in this critical early intervention period remains unacceptably low [1].

Low early treatment rates contribute significantly to physical, emotional and psychosocial impacts of eating disorders, which cost the Australian healthcare system $69 billion per year [2]. Anorexia Nervosa (AN) is associated with psychosocial disability and carer burden equal to that seen in chronic schizophrenia [3, 4]. Approximately five Australians die every day due to the effects of an eating disorder [2], while only one in four individuals seeks help for their condition [5]. Twenty percent of those who do seek help will go on to experience a chronic course [6], partially attributable to this paucity of initial help-seeking.

Prolonged illness duration raises mortality risk [7] and can be avoided with appropriate early identification and treatment provision [8]. Available evidence-based treatment for EDs is more effective if delivered early in the course of illness [9] highlighting the imperative to develop instruments that identify high risk and early stage or sub-threshold illness. Importantly, sub-threshold eating disorders are more likely to evolve into full-syndrome illness than to remit when untreated [10].

Identification of eating disorders is routinely achieved by lengthy self-report or clinician-administered diagnostic instruments which may not be suitable for time-poor physicians in primary care settings [11, 12]. Moreover, few existing tools reliably identify broad eating disorder presentations as defined by the newest iteration of the DSM [5] (adding to the diagnostic nomenclature Binge Eating Disorder (BED), Avoidant Restrictive Food Intake Disorder (ARFID), and Other Specified Feeding or Eating Disorder (OSFED). The most frequently used screening tool, the SCOFF questionnaire [13], typically identifies traditional presentations of AN and Bulimia Nervosa (BN) only [14]. This is despite higher population prevalence of binge eating and other specified eating disorders (3–6% of the population suffer from Binge Eating Disorder at any one time [15] and a further 7% subthreshold BED. 1–2% of the population suffers from AN [16]). Finally, existing screening and diagnostic tools utilise inflexible cut-off points for a disorder that emerges on a spectrum, and may not identify individuals at risk. They assume an individual has already presented to healthcare services at time of delivery, controverting what we know about accessibility and help-seeking delay in this population.

Novel screening tools should be designed to be embedded within the digital environment, particularly as this is the domain of those most at risk (young people) [17]. For an illness such as an eating disorder, often marked by stigma and lack of understanding of the complexity of associated psychopathology, digital tools present unprecedented opportunity to screen diverse populations early and can be utilised to inform and encourage ambivalent users to seek treatment. This is especially pertinent since the global pandemic of the SARS-CoV-2 virus drove a rapid and ongoing shift to digital healthcare delivery [18, 19]. Prior to the pandemic, 84% of Australians were seeking healthcare information online before presenting to primary care [20]—those numbers are likely higher now.

A number of unique risk factors contribute to increased probability of developing an eating disorder including perfectionism, negative affectivity and self-appraisal, and history of being bullied or teased [21]. Two of the most influential—dieting and body dissatisfaction— should be easily screened for but are going largely undetected in primary care. The 6-item digital InsideOut Screener (Table 1) was designed to assess for these risk factors as well as recognised features of eating disorder pathology. Co-designed with lived experience experts and clinicians, it is designed to assess eating disorder risk and subthreshold illness across the population. As a first point of contact for an illness marked by ambivalence, the screener may be used by individuals for self-identification and self-referral, or for rapid identification in clinical settings when delivered online. The purpose of this study was to statistically validate the instrument in adolescent and adult populations.

**Table 1:** Items of the InsideOut Institute Screener

Theme	Item
Relationship with food^a^	1. How is your relationship with food? *(For example: is food and eating worry free, or is it full of worry and stress?)*
Body & self-worth^a^	2. Does your weight, body or shape make you feel bad about yourself? *(For example: the number on the scale, the shape of your body or a part of your body.)*
Preoccupation with food or weight^a^	3. Do you feel like food, weight or your body shape dominates your life? *(For example: experiencing constant thoughts about food, weight or your body.)*
Anxiety and distress^b^	4. Do you feel anxious or distressed when you are not in control of your food? *(For example: when others cook or prepare food for you or when eating out.)*
Loss of control^c^	5. Do you ever feel like you will not be able to stop eating or have lost control around food? *(For example: feeling that you have no control around food, that you binge eat or fear that you will binge eat.)*
Compensatory behaviour^d^	6. When you think you have eaten too much, do you do anything to make up for it? *(For example: skipping the next meal, going light on the next meal, working it off with exercise, purging *via* vomiting or taking laxatives, diuretics or diet pills.)*

Items are rated on a 5-point Likert scale, where 1 is “never” and 5 is “all the time”; except for Question 1, where 1 is “worry and stress free” and 5 is “full of worry and stress”

^a^Relates to all presentations

^b^Relates to AN, BN and OSFED presentations

^c^Relates to BN, BED, and OSFED presentations

^d^Relates to AN-BP, BN, Purging Disorder and OSFED presentations

## Methods

### Study design and procedure

Methodological protocol including item generation, study design, sample size estimations, explanation of projected statistical analyses, and description of the way in which the screening tool is utilised, has been previously published in full [22]. The present study employed a mixed cross-sectional and repeated measures longitudinal survey research design. Individuals aged 14 and over who read English were recruited via Mechanical Turk, social media and through flyer advertising in clinical settings. They completed a comprehensive survey battery designed to assess standard measures of ED symptom severity and statistical reliability and validity. 254 (19%) completed the IOI-S a second time two weeks post initial testing, for test–retest reliability analysis. A two-week interval is commonly employed in test–retest reliability analysis as it is short enough to prevent change in true score, but not so short to violate independence due to recall [23–25]. This study was approved by the University of Sydney Human Research Ethics Committee (HREC) (Protocol No. 2020/363). The authors followed the Standards for Reporting of Diagnostic Accuracy Studies 2015 (STARD) guidelines [26].

### Measures

The baseline survey battery consisted of the IOI-S, the extensively validated Eating Disorders Examination Questionnaire [27], its 8-item short form the EDE-Q8 [28], the SCOFF screening questionnaire [13), and one of two measures of social desirability—the Marlowe Crowne Social Desirability Scale [29] or the Children’s Social Desirability Scale [30].

## InsideOut Institute Screener (IOI-S) (InsideOut Institute for Eating Disorders) (2018) [31]

Item development followed review of the scientific literature for existing instruments screening and assessing eating disorder symptomatology, lived experience and clinical and research expert consultation. Instruments reviewed included the EDE-Q, the Eating Disorders Inventory (Garner et al.,), the SCOFF questionnaire, the ESP, and the Eating Attitudes Test (EAT; Garner et al.,), from which researchers developed an initial pool of 10 questions covering six facets of eating pathology: an individual’s relationship with food, body, the extent to which body weight and shape determines self-worth, loss of control over eating, binge eating and compensatory behaviour. This was further narrowed by an expert consultation team to six relevant items to evaluate eating pathology and eating disorder risk in a non-clinical population. The IOI-S is rated on a 5-point Likert scale, where 1 is “never” and 5 is “all the time”; except for Question 1, where 1 is “worry and stress free” and 5 is “full of worry and stress”. These items do not refer to a particular timeframe, e.g., the previous 28 days; rather they are about how an individual typically feels and are designed to “start a conversation”. Responses are summed to yield a score between 6 and 30 points total, where 6 points is the lowest degree of risk and 30 points the highest degree of risk. The screener is currently available on the InsideOut Institute website behind a prompt “are you at risk?” and is wholly self-directed. Those deemed to be of moderate to high risk according to score are directed to a database of trained professionals with expertise in eating disorders and encouraged to seek help. Designed to be embedded into existing healthcare structures, the screener is currently being validated for face-to-face use in primary care settings.

## Eating Disorder Examination Questionnaire (EDE-Q) (Fairburn & Beglin) [27]

The EDE-Q is a 28-item self-report version of the EDE structured clinical interview which is regarded as the ‘gold-standard’ diagnostic self-report tool in eating disorders and has been validated in multiple trials. Traditionally paper-based, it has been psychometrically validated for online delivery. It includes additional measures of weight, height, and missed menstrual periods, the latter of which was excluded from our study due to the DSM-5 removal of and consequent diagnostic irrelevance of missed menstruation in ED. It is comprised of a global score and four subscales: Restraint, Eating Concern, Weight Concern, and Shape Concern, and employs a 7-point forced-choice severity rating where 0 points is the lowest severity and 6 points is the highest severity.

## SCOFF (Sick, Control, One stone, Fat, Food) Questionnaire (Morgan et al.) [13]

The SCOFF questionnaire is a short 5-item forced choice (true/false) screening tool designed to assess eating disorder symptomatology. A threshold of > 2 positive answers indicates a ‘likely case’ of AN or BN. It has shown good internal consistency and concurrent validity with the EDE-Q. The SCOFF is included due to the frequency with which it has been employed in clinical and research settings.

## Eating Disorder Examination Questionnaire 8 (EDE-Q8) (Kliem et al.) [28]

The EDE-Q8 is an 8-item short form of the longer EDE-Q, designed as an abbreviated outcome measure that retains the original factor structure. It is highly correlated with the EDE-Q and demonstrates strong internal consistency, concurrent and convergent validity. Items are drawn directly from and are identical to respective items in the EDE-Q and were extracted for independent analysis in this instance.

## Marlowe-Crowne Social Desirability Scale (MC-SDS) (Crowne & Marlowe) [29]

The MC-SDS is a well-validated 33-item self-report questionnaire measuring social desirability in adults by evaluating concern with social approval. Items are rated true/false and balanced for positive and negative wording. It is frequently used as a measure of discriminant validity in instrument design and has shown good divergent validity with measures of psychopathology (anxiety, depression) related to eating disorders. The MC-SDS is not validated for use in children: thus, an adapted Children’s Social Desirability Scale (CSD-S) applied for those participants aged under 18.

## Children’s Social Desirability Short Scale (CSD-S) (Baxter et al.) [30]

The CSD-S is an abbreviated 14-item version of the gold-standard 48-item Children’s Social Desirability scale developed by Crandall et al. in 1965 [32]. The original scale was modelled on the MC-SDS for adults and is validated for use in a child/adolescent population. The CSD-S was assigned to our 14 to 17-year-old participants. The scale uses binary response (yes/no) and is regularly used for methodological validity to detect confounding from social desirability bias. The CSD-S has demonstrated adequate internal consistency and test–retest reliability and good external validity.

### Participants

Participants were divided into ‘probable ED’ and ‘healthy’ groups post initial data analysis based on an EDE-Q global score of 2.3. The EDE-Q global score (range 0–6) is used widely in clinical practice to assess eating disorder status, with threshold recommendations ranging from approximately 2–4 [33–35]. For the purposes of this study, participants scoring 2.3 or above [36] were considered ‘probable ED’ and those scoring below 2.3 were deemed healthy.

### Outcome

The main outcome was probable eating disorder diagnosis determined by an EDE-Q score of 2.3 and satisfaction of diagnostic behavioural criteria, or deemed moderate or high risk (sub-threshold) eating disorder determined by symptomatic clinical algorithm adapted from Berg et al. [37] (see Additional file 1). A cut-off of 2.3 was chosen for ROC curve analysis based on previous literature [36], clinical experience and independent data analysis.

### Statistical analyses

Factor analysis was first conducted to ensure unidimensionality had been met [38]. Data was then analysed in three sequential stages to assess measure reliability, validity and sensitivity/specificity.

All 6 items of the IOI-S were expected to load onto a single factor. Principal axis factoring was chosen to determine factor structure because it deals with latent dimensions and does not assume normative distribution [39]. We used Kaiser criterion to guide factor decisions, which suggests factors with eigenvalues of > 1.00 are common and should be retained [40].

Measure reliability of the IOI-S was examined using internal consistency, or the degree to which the six items of the scale measure the same underlying dimension [41] (Cronbach’s alpha) and test–retest reliability, or the correlation between successive iterations of the test [42] (Intraclass Correlation Coefficient). As data was non-parametric, Spearman’s rank order correlation was appropriate to assess for concurrent, convergent and discriminant validity [43].

Receiver Operating Characteristic (ROC) curve analysis was performed to examine sensitivity and specificity, or the ability of the IOI-S to accurately identify true positive and true negative cases [44] and to establish two sub-thresholds indicating moderate and high risk of developing an eating disorder. Categorical criteria for DSM-5 diagnoses were operationalised using established clinical algorithm [37]. Data was analysed to divide subthreshold participants into two groups: those at high risk based on sub-clinical behaviours and high attitudinal scores (these participants scored between 1.8 and 2.3 on the EDE-Q); and those at moderate risk based on minimal behaviours and moderate attitudinal scores (these participants scored between 1.3 and 1.8 on the EDE-Q). All data were analysed in SPSS v.27.

### Additional correlations between IOI-S and measures of binge eating

The EDE-Q was conceived prior to the addition of Binge Eating Disorder to the diagnostic nomenclature, and existing screeners often fail to adequately identify this presentation [45]. As such, binge eating pathology was analysed separately for concurrent validity. Research shows the eating concern subscale and item 15 (OBE’s) to be most representative of the EDE-Q’s ability to identify Binge Eating Disorder [46]. These were extracted for independent analysis against the performance of both the IOI-S item relating to binge eating (item 5) and the total IOI-S score.

## Results

There was no missing scalable data: 1346 participants completed all items except the items concerning weight and height. 1236 participants completed these items, which were not included in any statistical analyses: they were only operationalised to produce specific diagnostic information. Mean scores on the two primary measures are presented in Additional file 2.

### Participant characteristics

1346 participants aged 14–74 (*M* = 26.60, *SD* = 11.14) completed the baseline survey battery between July 2020 and February 2021: 304 (22.6%) male, 993 (73.8%) female, and 49 (3.6%) identifying as ‘other’ (see Table 2). 71.1% (*n* = 957) met the cut-off for a probable eating disorder based on an EDE-Q global cut-off score of 2.3. Females were more likely to have a probable eating disorder (78%) than were males (46.4%). Participants were predominantly Caucasian, *n* = 1047 (77.8%) followed by Asian, *n* = 175 (13.0%) and Hispanic *n* = 27, (2.0%). Mean self-reported BMI was 23.97, *SD* = 6.05 (10.67–56.61).

**Table 2:** Participant demographic characteristics

	Healthy (*n* = 389)	Probable ED (*n* = 957)	Total (*n* = 1346)
*Gender n (%)*			
Female	218 (21.9)	775 (78.0)	993 (73.8)
Male	163 (53.6)	141 (46.4)	304 (22.6)
Other	7 (14.3)	42 (85.7)	49 (3.6)
Total n (%)	389 (28.9)	957 (71.1)	1346 (100)
*Ethnicity n (%)*			
ATSI	2 (10.5)	17 (89.5)	19 (1.4)
Caucasian	285 (27.2)	762 (72.8)	1047 (77.8)
Asian	75 (42.9)	100 (57.1)	175 (13.0)
Middle Eastern	5 (35.7)	9 (64.3)	14 (1.0)
Pacific Islander	1 (25.0)	3 (75.0)	4 (0.3)
Hispanic	4 (14.8)	23 (85.2)	27 (2.0)
African	9 (52.9)	8 (47.1)	17 (1.26)
Other	7 (16.3)	36 (83.7)	43 (3.2)
*Total n (%)*	389 (28.9)	957 (71.1)	1346 (100)

Of the participants meeting the EDE-Q global score cut-off of 2.3, the majority presented as likely OSFED Atypical AN, *n* = 246 (18.3%). Likely BN was next most common, *n* = 151 (11.2%), followed by Binge Eating Disorder, *n* = 126 (9.4%) and Unspecified Feeding or Eating Disorder, *n* = 94 (7.0%) (Additional file 3).

### Factor analysis

A Kaiser-Meyer Olkin measure of sampling adequacy of 0.911 and a significant Bartlett’s test of sphericity (*χ*^2^ [15] = 5128.02, *p* < 0.001) found the screener to be factorable and thus suitable for structure detection. One factor was retained, with an Eigenvalue of 3.787, accounting for approximately 63.11% of the total variance observed (Additional file 4). Given the single retained factor, rotation was not executed. Parallel analysis was conducted for corroboration. Using O’Connor’s syntax [47] we compared eigenvalues generated by principal components analysis with randomly generated eigenvalues and found that factors with an eigenvalue of 1.00 or above should be retained, producing the same one-factor solution.

### Reliability of the IOI-S

The 6 items of the scale exhibited strong internal consistency (α = 0.908) (Additional file 5). Cronbach’s alpha did not exceed 0.908 when any individual item was deleted: therefore, the use of all items together yields optimal internal consistency. We additionally ran McDonald’s omega analysis using Hayes Omega macro [48]. This demonstrated excellent scale reliability (ω = 0.910).

50% of individuals were randomised to complete the two-week re-test, with a completion rate of 19% (245 participants). Mean estimations and 95% confidence interval were reported. The Intraclass Correlation Coefficient between successive iterations of the test was significantly positive, being 0.968 with a 95% confidence interval from 0.959–0.975 (F(253, 253) = 31.44, *p* < 0.01 (see Table 3).

**Table 3:** InsideOut Institute Screener Test–retest reliability analysis

	*n*	Intraclass correlation	95% confidence interval		F Test with True Value 0			
			Lower bound	Upper bound	Value	*df*1	*df*2	Sig
Average measures	254	.968	.959	.975	31.438	253	253	.000

Test–Retest Reliability Analysis using 2-way mixed-effects model Intraclass Correlation Coefficient, where people effects are random and measure effects are fixed. Re-test of the IOI-S was conducted two weeks post initial testing

### Validity of the IOI-S

#### Concurrent validity

Concordance between the IOI-S and established measures of eating disorder symptomatology the EDE-Q, the EDE-Q8 and the SCOFF was assessed using a Spearman’s Rank Order correlation (see Table 4). Bivariate correlation analysis found the IOI-S to be strongly positively correlated with the EDE-Q, *r*_*s*_ 0.88 (*p* < 0.01), the EDE-Q8, *r*_*s*_ 0.84 (*p* < 0.01), and the SCOFF questionnaire, *r*_*s*_ 0.75 (*p* < 0.01). Of the secondary measures, the SCOFF demonstrated the weakest overall correlation with the EDE-Q, *r*_*s*_ 0.67 (*p* < 0.01).

**Table 4:** Spearman’s correlations for concurrent and discriminant validity

*n* = 1346	Correlation coefficient	Sig (2-tailed)**
IOI-S/EDE-Q	.885**	.000
IOI-S/EDE-Q8	.838**	.000
IOI-S/SCOFF	.749**	.000
EDE-Q/EDE-Q8	.964**	.000
EDE-Q/SCOFF	.671**	.000
IOI-S/MC-SDS (*n* = 1019)	− .087**	.005
IOI-S/CSD-S (*n* = 327)	.010	.859
*Binge item analysis*		
IOI-S Item 5/EDE-Q item 15	.533**	.000
IOI-S Item 5/EDE-Q eating	.635**	.000
IOI-S Total/EDE-Q item 15	.349**	.000
IOI-S Total/EDE-Q eating	.855**	.000
EDE-Q Item 15/EDE-Q eating	.364**	.000
EDE-Q global/EDE-Q item 15	.255**	.000
EDE-Q global/EDE-Q Eating	.918**	.000

EDE-Q Item 15 = OBEs (objective binge episodes); eating = eating concern subscale; IOI-S Item 5 = loss of control

**Correlation is significant at the 0.01 level [2-tailed]

#### Convergent Validity

The IOI-S was designed to measure the same broad symptomatology as the EDE-Q and SCOFF questionnaires and as such, it significantly converged with both (0.88 *p* < 0.01 and 0.75 *p* < 0.01 respectively). At the item level, Spearman’s correlations showed moderate to strong convergence between IOI-S variables (Additional file 6). All inter-item correlations were significant, with items 1 and 3 demonstrating the strongest convergence, 0.733 (*p* < 0.01). Items 5 and 6 demonstrated the weakest inter-item convergence, 0.460 (*p* < 0.01), but the relationship was still significant.

#### Discriminant validity

A scale with good discriminant validity will have scores that are unassociated or negatively associated with scores on a scale measuring unrelated or opposing constructs [49].

A bivariate Spearman’s correlation analysis showed a significant negative correlation between the IOI-S and the Marlowe Crowne Social Desirability Scale, *r*_*s*_ = -0.087 (*p* < 0.01) (Table 4). There was no significant relationship between the IOI-S and the Children’s Social Desirability Scale, *r*_*s*_ 0.010 (*p* > 0.01), but it nevertheless diverged.

#### Independent binge item analysis

On measures of binge eating, the full IOI-S demonstrated a strong positive correlation with the EDE-Q Eating Concern Subscale, 0.85 (*p* < 0.01) (Table 4). IOI-S item 5 showed a moderate-strong positive correlation with the EDE-Q Eating Concern subscale, 0.63 (*p* < 0.01), and a moderate positive correlation with the EDE-Q item concerning objective binge episodes, 0.53 (*p* < 0.01).

#### Sensitivity and specificity

**Fig. 1:**
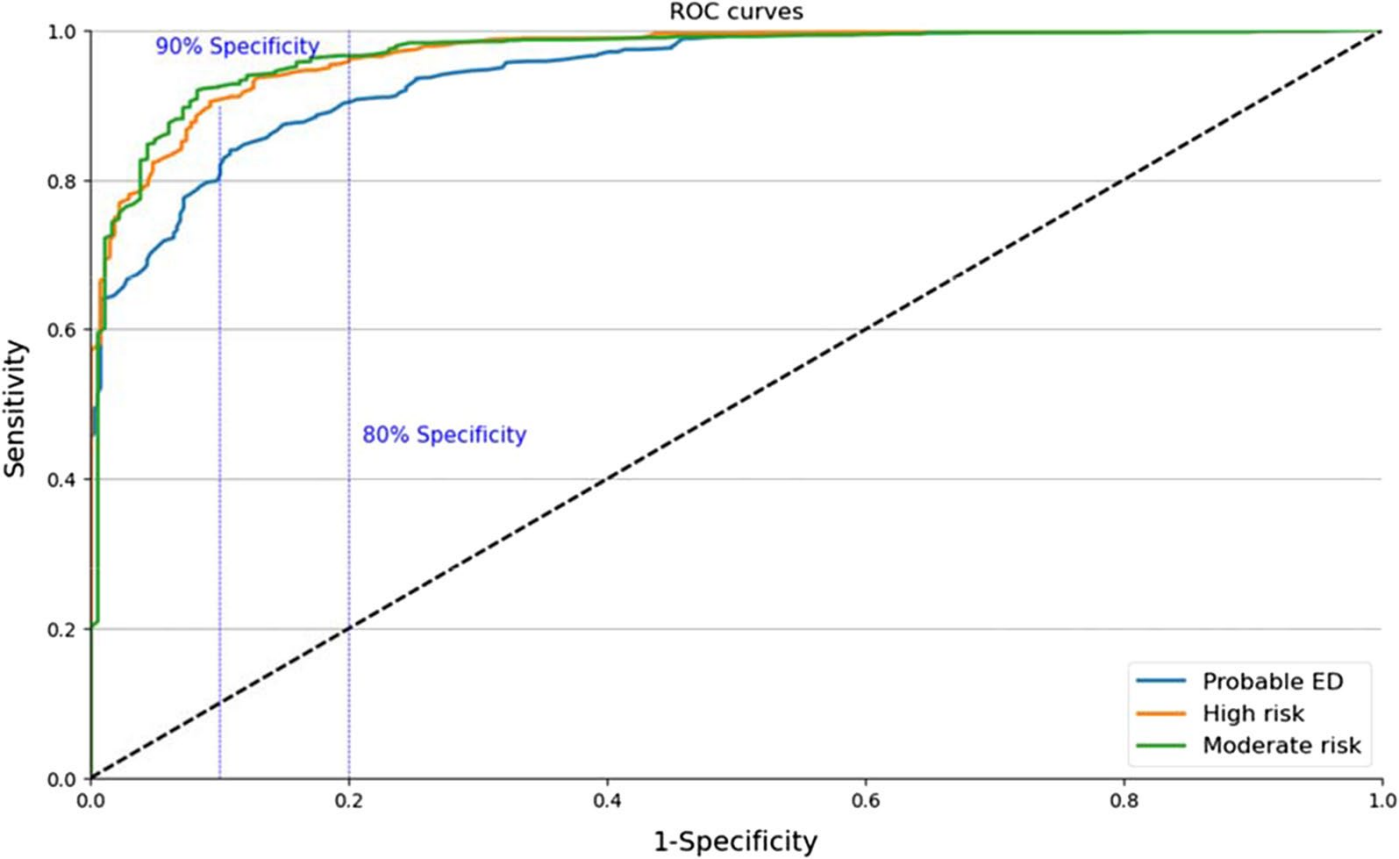
Receiver operating characteristic curves for the InsideOut Institute Screener (IOI-S) at Probable ED, Moderate and High Risk Thresholds

The AUC for application of IOI-S algorithms was excellent at all three levels (see Fig. 1). IOI-S cut off of 18.50 AUC = 0.944 (95% CI, 0.932–0.955) demonstrated the highest diagnostic capability for distinguishing between eating disordered and healthy cases with sensitivity of 82.8% and specificity of 89.7%. The SCOFF questionnaire was analysed for sensitivity and specificity on the same threshold of 2.3, using a syndromal SCOFF score of 2.5 (two or more positive answers indicates a likely eating disorder [13]. Both sensitivity (65.4%) and specificity (82.7%) were markedly lower than for the IOI-S, as was overall Area Under Curve (AUC = 0.833).

ROC Curve analysis was performed on two further EDE-Q global score cut-offs in order to determine appropriate IOI-S thresholds for moderate and high-risk individuals (Table 5). At 1.8, or ‘high’ risk, ROC curve analysis yielded a parallel IOI-S cut off of 15.50 (AUC = 0.970, 95% CI, 0.961–0.979), with good sensitivity (90.3%) and specificity (90.7%). At 1.3, or ‘moderate’ risk, ROC curve analysis yielded a parallel IOI-S cut off of 13.50 (AUC = 0.969, 95% CI, 0.957–0.981), with excellent sensitivity (91.9%) and specificity (91.8%).

**Table 5:** Receiver Operating Curve Results (IOI-S Sensitivity and Specificity)

Area under the curve			Coordinates of the curve		
Threshold	Area	Sig	Positive if >	Sensitivity	1-Specificity
Probable ED	.944	.000	18.50	.828	.103
High risk	.970	.000	15.50	.903	.093
Moderate risk	.969	.000	13.50	.919	.082

The vertical lines indicate the 80% and 90% specificity cut points.

## Discussion

Reliable detection of emerging eating disorder behaviour is critical for strategic early intervention and may significantly impact an individual’s illness course and risk of mortality. Evidence-based treatments for the most life-threatening eating disorders are more effective if delivered early in the course of illness [9, 50], thus early identification of risk and symptomatology is imperative. Results provide excellent initial support for the psychometric validity of the IOI-S in a broad age range of all genders. Designed specifically for online use, it is validated for the platform on which the most at-risk population seeks healthcare information; is scalable within different health contexts and is suitable for self-directed treatment seeking.

The instrument demonstrated excellent psychometric properties. Each item of the IOI-S measured the same underlying dimension and a strong positive correlation was found between baseline and repeated testing. Thus, the IOI-S demonstrates utility beyond early intervention, with potential to be administered repeatedly throughout treatment as a measure of ongoing or recovered symptomatology.

The IOI-S correlated strongly with the well-utilised self-report diagnostic instrument the EDE-Q and its brief form derivative the EDE-Q8. It outperformed the SCOFF questionnaire on correlation with the EDE-Q, providing support for our overarching aim to improve screening capacity for a range of eating disorder presentations. The IOI-S also performed better than the SCOFF questionnaire on sensitivity and specificity, where the latter’s specificity was markedly reduced. That is, the SCOFF was more likely to assign false positive status to those who were not actually ill, as has been found in previous studies [14, 35, 51]. Further, the IOI-S benefits from a range of features missing from the SCOFF that enhance its utility for early identification: it is digitally designed and validated, scalable, utilises self-report and employs non-confronting language.

The importance of developing tools to improve identification and referral of Binge Eating Disorder has been well documented [52, 53]. The present study is one of few that have looked at and been validated against EDE-Q binge eating items independently of global score. Both the IOI-S total score and item 5 alone were better able to capture problematic binge eating behaviour than was the EDE-Q global score, providing support for the screener’s ability to identify presentations of eating disorder beyond typical AN and BN.

The ability of the screener to assign risk thresholds to subsyndromal and early presentations in addition to correctly distinguishing ill from not ill, was an important aim of the present study. High sensitivity and specificity on all three thresholds suggest the tool may be reliably used to discern risk and degrees of symptomatology. Importantly, considerable contention exists within the expert community over the stringency of current full-syndrome criteria for the major eating disorders, which may exclude or prevent individuals experiencing earlier stage illness from receiving treatment. This strengthens the need for mechanisms to capture sub-threshold and early cases, as the IOI-S does. Early intervention efforts may be strengthened by identifying those at risk, rather than considering only those who are ‘definitely ill’ in the provision of interventions and services. This may have significant implications for individual illness trajectory, overall healthcare burden and mortality rates.

The methodological design of the present study enabled recruitment of a large pool of participants of relatively diverse ethnicity, age, clinical presentation and gender, greatly increasing generalisability. Relative to existing assessment literature—and eating disorder research in general—the rate of male participants in this sample was very high. BMI, gender and age were all reported. Participants who were female were more likely to display eating disorder symptomatology, which was not unexpected in an illness group that remains predominantly female [54, 55].

### Limitations

Validity is not an immutable property: it is a function of participant or patient characteristics, diagnostic categories and testing environments. A large percentage of individuals recruited into this study were identified as having a probable ED. This may be a function of the targeted nature of the social media advertising, which used algorithms to identify individuals displaying interest in content relating to eating disorders; as well as traditional advertising in the form of flyers displayed in clinical eating disorder settings. Research should be undertaken in confirmed clinical and non-clinical populations to better assess the IOI-S’ ability to distinguish ill from healthy and to identify diagnostic subtypes.

Research shows First Australians and those of ethnic minority experience higher incidence of eating disorders [56, 57] and this was supported by our results. Those identifying as Aboriginal or Torres Strait Islander, Hispanic, African or Middle Eastern were much more likely to be symptomatic than were Caucasians, Asians or Pacific Islanders. Despite efforts to attract a broad demographic to the current study, generalisability to non-Caucasian cultures will require further study. To a lesser extent, further examination across all genders is warranted.

### Conclusions

Eating disorders are complex psychological conditions. Evidence-based treatment and prevention exists and is effective but is not delivered to many people, and often late in the illness trajectory. This is confounded by lack of physician training and inappropriate or arduous primary screening measures. To reduce the burden of disease associated with eating disorders it is vital to intervene prior to or soon after onset and the InsideOut Institute Screener is valid to this effect, offering individuals and clinicians a sensitive, stepped screening approach suitable for self-administration, easily scalable across primary care settings, and consistent with the digital era.

## Supplementary Information

**Additional file 1.** Diagnostic Algorithms for EDE-Q.

**Additional file 2.** IOI-S and EDE-Q Participant Mean Scores.

**Additional file 3.** Participant DSM-5 Eating Disorder Diagnostic Characteristics.

**Additional file 4.** Factor Loadings for the 6 Survey Items.

**Additional file 5.** InsideOut Institute Screener Internal Consistency Analysis.

**Additional file 6.** InsideOut Institute Screener Inter-Item Correlations.

## Data Availability

Not applicable.

## Abbreviations

AN: Anorexia Nervosa
ARFID: Avoidant Restrictive Food Intake Disorder
BED: Binge Eating Disorder
BN: Bulimia Nervosa
ED: Eating Disorder
EDE-Q: Eating Disorder Examination Questionnaire
EDE-QS: Eating Disorder Examination Questionnaire Short
IOI-S: InsideOut Institute Screener
OSFED: Other Specified Feeding or Eating Disorder
SCOFF: Sick, Control, One Stone, Fat Food questionnaire
MC-SDS: Marlowe-Crowne Social Desirability Scale
CSD-S: Children’s Social Desirability Short Scale
