# Supplementary material for: Identification of high risk and early stage eating disorders: first validation of a digital screening tool
Source: J Eat Disord. 2021 Sep 6;9:109. doi: 10.1186/s40337-021-00464-y (PMC8419810; doi:10.1186/s40337-021-00464-y)
Supplement: Supplementary file 1 — Additional file 1. Diagnostic Algorithms for EDE-Q. [file 40337_2021_464_MOESM1_ESM.docx]

**Additional File 1.** *Diagnostic algorithms for EDE-Q***

| **DSM-5 criteria** | **EDE-Q criteria** |
| --- | --- |
| **Anorexia Nervosa** |  |
| a. Restriction leading to low body weight | Items 1-3 >3; %IBW<85% |
| b. Fear of weight gain | Item 10 >3; or compensatory behaviours > once per week |
| c. Body image disturbance | Weight subscale >2.3; or Shape subscale >2.3 |
| d. Restricting type (R)  Binge/purge type (BP) | Item 13-17 <1  Item 13-17 >1 |
| **Bulimia Nervosa** |  |
| a. Binge eating | OBEs endorsed |
| b. Compensatory behaviour | Vomiting, laxative or diuretic use endorsed |
| c. Frequency/duration^a^ | Total OBEs > once per week (item 15 >4)  and total compensatory behaviours > once per week (item 16 or 17 or 18 >4). |
| d. Over-evaluation of shape or weight | Weight subscale >2.3; or Shape subscale >2.3 |
| e. Not AN | %IBW>85% |
| **Binge Eating Disorder** |  |
| a. Binge eating | OBEs endorsed |
| b. Associated features^b^ | Item 14>4 |
| c. Distress^c^ | Item 9>2 |
| d. Frequency/duration^a^ | Total OBEs > once per week (item 15 >4) |
| e. No regular compensatory behaviours/not AN or BN | Total compensatory behaviours < once per month (item 16 or 17 or 18 <1) and %IBW>85% |
| **OSFED Atypical AN** |  |
| a. All criteria met for AN except criteria a (low body weight) | IBW > 85% |
| **OSFED BN low frequency** |  |
| a. All criteria met for BN  except binge eating and  compensatory behaviour  occurs infrequently. | All criteria met for BN except total OBEs < less than once per week (item 15 <4); total compensatory behaviours < once per week (item 16 or 17 or 18 <4) |
| **OSFED BED low frequency** |  |
| a. All criteria met for BED  except binge eating  behaviour occurs  infrequently. | All criteria met for BED except total OBEs < once per week (item 15 <4) |
| **OSFED Other** |  |
| a. Clinically significant eating  disorder that either does not meet full criteria for any  individual diagnosis or  crosses several diagnoses. | – |
| **OSFED Purging Disorder** |  |
| a. Recurrent purging  behaviour in the absence of  binge eating. | Total OBEs < once per month (item 15 <1); item 16 >4 |
| **UFED** |  |
| a. Clinically significant eating  disorder without sufficient  information to justify  specific diagnosis. | Clinically significant eating disorder as decribed elsewhere but no self-reported weight or height (BMI missing) |
| **Does not meet criteria** |  |
| a. No clinically significant ED  cognitions or behaviours  despite meeting global score  cut off | None of above criteria met |

AN = anorexia nervosa; Atyp. AN = atypical anorexia nervosa; BN = bulimia nervosa; BED = binge eating disorder; BMI = body mass index; ED = eating disorder; IBW = ideal body weight; OSFED = other specified feeding or eating disorder; UFED = unspecified feeding or eating disorder; OBE = objective binge episode.

^a^Over 28 days rather than three months as stipulated in DSM-5

^b^Feeling of loss of control over eating

^c^Clinically significant distress as based on Item 9 – fear of losing control over eating

**Clinical diagnoses estimates based on EDE-Q self-report and scoring algorithm adapted from Berg et al., 2012

*Note.* Some DSM-5 diagnostic categories such as ARFID were not able to be assessed due to the inability of the EDE-Q to capture behavioural or attitudinal features associated with these disorders.
