# Supplementary material for: Identification of high risk and early stage eating disorders: first validation of a digital screening tool
Source: J Eat Disord. 2021 Sep 6;9:109. doi: 10.1186/s40337-021-00464-y (PMC8419810; doi:10.1186/s40337-021-00464-y)
Supplement: Supplementary file 2 — Additional file 2. IOI-S and EDE-Q Participant Mean Scores. [file 40337_2021_464_MOESM2_ESM.docx]

**Additional File 2.** *IOI-S and EDE-Q participant mean scores*

| ***n* = 1346** | **IOI-S** | **EDE-Q** |  |  |  |  |
| --- | --- | --- | --- | --- | --- | --- |
| **Subscales** |  | **Restraint** | **Eating** | **Shape** | **Weight** | **Global** |
| **Range** | 6 – 30 | 0 – 6 | 0 – 6 | 0 – 6 | 0 – 6 | 0 – 6 |
| **Mean (M)** | 19.86 | 2.88 | 2.60 | 3.86 | 3.69 | 3.26 |
| **Standard Deviation (SD)** | 6.58 | 1.85 | 1.71 | 1.68 | 1.66 | 1.58 |

*Note.* Mean scores from two self-report questionnaires (the InsideOut Institute Screener and the Eating Disorder Examination Questionnaire) delivered online at one time-point.
