# Supplementary material for: Identification of high risk and early stage eating disorders: first validation of a digital screening tool
Source: J Eat Disord. 2021 Sep 6;9:109. doi: 10.1186/s40337-021-00464-y (PMC8419810; doi:10.1186/s40337-021-00464-y)
Supplement: Supplementary file 3 — Additional file 3. Participant DSM-5 Eating Disorder Diagnostic Characteristics. [file 40337_2021_464_MOESM3_ESM.docx]

**Additional File 3.** *Participant DSM-5 eating disorder diagnostic characteristics*

| **Total *n* = 957** | **(n) (%)** |
| --- | --- |
| OSFED Atypical AN | 246 (25.7) |
| BN | 151 (15.8) |
| BED | 126 (13.2) |
| UFED | 94 (9.8) |
| OSFED Other | 79 (8.2) |
| AN-BP | 66 (6.9) |
| OSFED BN low freq. | 58 (6.1) |
| AN-R | 50 (5.2) |
| OSFED BED low freq. | 46 (4.8) |
| No criteria met | 37 (3.9) |
| OSFED Purging disorder | 4 (0.4) |
| **Total** | **957 (100%)** |

AN = anorexia nervosa; AN-BP = anorexia nervosa binge purge subtype; AN-R = anorexia nervosa restrictive subtype; BMI = body mass index; BN = bulimia nervosa; BED = binge eating disorder; ED = eating disorder; OSFED = other specified feeding or eating disorder; UFED = unspecified feeding or eating disorder; “low freq.” = low frequency.

^**^Clinical diagnoses estimates based on EDE-Q self-report and scoring algorithm adapted from Berg et al., 2012
