# Supplementary material for: Identification of high risk and early stage eating disorders: first validation of a digital screening tool
Source: J Eat Disord. 2021 Sep 6;9:109. doi: 10.1186/s40337-021-00464-y (PMC8419810; doi:10.1186/s40337-021-00464-y)
Supplement: Supplementary file 4 — Additional file 4. Factor Loadings for the 6 Survey Items. [file 40337_2021_464_MOESM4_ESM.docx]

**Additional File 4.** *Factor Loadings for the 6 Survey Items*

| **Item** | **Factor 1** |
| --- | --- |
| Item 3. Preoccupation with food or weight | .888 |
| Item 4. Anxiety & distress around food | .871 |
| Item 1. Relationship with food | .826 |
| Item 2. Body & self-worth | .812 |
| Item 6. Compensatory behaviour | .675 |
| Item 5. Loss of control when eating | .666 |
| **Eigenvalues** | **3.787** |
| **% of variance** | **63.11** |

Extraction method: Principal Axis Factoring

*Note.* Items listed from highest to lowest loading
