# Supplementary material for: Identification of high risk and early stage eating disorders: first validation of a digital screening tool
Source: J Eat Disord. 2021 Sep 6;9:109. doi: 10.1186/s40337-021-00464-y (PMC8419810; doi:10.1186/s40337-021-00464-y)
Supplement: Supplementary file 5 — Additional file 5. InsideOut Institute Screener Internal Consistency Analysis. [file 40337_2021_464_MOESM5_ESM.docx]

**Additional File 5.** *InsideOut Insitute Screener Internal Consistency Analysis*

| ***n* = 1346** | **Cronbach’s *α*** | **Corrected item-total correlation** | **Cronbach’s *α* if item deleted** |
| --- | --- | --- | --- |
| **IOI-S total** | .908 |  |  |
| **Item 1** |  | .776 | .886 |
| **Item 2** |  | .764 | .889 |
| **Item 3** |  | .832 | .878 |
| **Item 4** |  | .821 | .879 |
| **Item 5** |  | .637 | .907 |
| **Item 6** |  | .645 | .905 |

*Note.* Corrected item-total correlation = correlation between item and the scale as a whole if item is deleted.
