# Supplementary material for: Identification of high risk and early stage eating disorders: first validation of a digital screening tool
Source: J Eat Disord. 2021 Sep 6;9:109. doi: 10.1186/s40337-021-00464-y (PMC8419810; doi:10.1186/s40337-021-00464-y)
Supplement: Supplementary file 6 — Additional file 6. InsideOut Institute Screener Inter-Item Correlations. [file 40337_2021_464_MOESM6_ESM.docx]

**Additional File 6.** *InsideOut Institute Screener Spearman’s Inter-Item Correlations (Convergent Validity)*

| ***n* = 1346** | **Item 1** | **Item 2** | **Item 3** | **Item 4** | **Item 5** | **Item 6** |
| --- | --- | --- | --- | --- | --- | --- |
| Item 1 | 1.00 | .683^**^ | .733^**^ | .730^**^ | .533^**^ | .528^**^ |
| Item 2 | .683^**^ | 1.00 | .729^**^ | .661^**^ | .506^**^ | .501^**^ |
| Item 3 | .733^**^ | .729^**^ | 1.00 | .733^**^ | .573^**^ | .587^**^ |
| Item 4 | .730^**^ | .661^**^ | .733^**^ | 1.00 | .582^**^ | .599^**^ |
| Item 5 | .533^**^ | .506^**^ | .573^**^ | .582^**^ | 1.00 | 4.60^**^ |
| Item 6 | .528^**^ | .501^**^ | .587^**^ | .599^**^ | .460^**^ | 1.00 |

^**^Correlation is significant at the 0.01 level (2-tailed)
